# A hybrid PSO-AVOA framework for patient-reported drug prioritization with enhanced exploration and exploitation

**DOI:** 10.3389/fdgth.2025.1708730

**Published:** 2025-11-12

**Authors:** Manickavasagam Suruthi, Narayanan Ganesh

**Affiliations:** School of Computer Science and Engineering, Vellore Institute of Technology, Chennai, India

**Keywords:** hybrid optimization, particle swarm optimization (PSO), enhanced African vulture optimization algorithm (EAVOA), drug prioritization, clinical decision support systems (CDSS), precision medicine

## Abstract

**Introduction:**

Patient-generated drug reviews are becoming increasingly available and serve as a rich source for computational drug prioritization.

**Methods:**

In this study, we developed a Hybrid Particle Swarm-Enhanced African Vulture Optimisation Algorithm (Hybrid PSO-EAVOA) that fosters the development of better balances between the exploration and exploitation of which the framework uses the improved opposition-based learning, Levy flights, and elite preservation approaches. In the framework, multiple evaluation criteria are accommodated, recovering value in the form of an overall single-objective optimization scheme, where effectiveness, side-effects, and consistency of reviews were compiled for clinical significance and combined by a weighted-sum fitness function. To validate the experiment using a large-scale dataset of drug reviews obtained from the Drugs Side Effects and Medical Condition dataset sourced from Drugs.com in Kaggle.

**Results:**

Hybrid PSO-EAVOA performed a benchmark comparison against five state-of-the-art metaheuristic algorithms (PSO, EAVOA, WHO, ALO, and HOA) using varying iterations as runs. In each comparison, Hybrid PSO-EAVOA achieved superior or better convergence speed, robustness, and quality of solutions.

**Discussion:**

The specific method of weighted-sum aggregation was used in this study, the framework offered could be easily compatible with other forms of aggregation. Hybrid PSO-EAVOA demonstrates strong potential for broader application in fields such as pharmacovigilance, clinical decision support, and drug re-purposing. The dataset is publicly available on Kaggle Drugs Side Effects and Medical Condition and all source code for parameter settings and preprocessing scripts is publicly available at the GitHub repository https://github.com/suruthi-m/Hybrid_PSO_EAVOA.

## 1 Introduction

Personalized healthcare is evolving and data-driven, intelligent decision-support systems have become increasingly important. Electronic health records, user-sourced reviews and metadata regarding drugs provide many opportunities and challenges in optimizing drug selection (1). Developing personalized medication decisions requires assessing several variables, including efficiency, side effects, comorbidities, demographic characteristics and user notes. Rule-based (or simple statistical model) approaches typically will not work well for the complexities and high dimensionality of real-world clinical scenarios, especially with incomplete or heterogeneous data sources (2, 3). This complexity has created interest in applying artificial intelligence (AI) and nature-inspired metaheuristics algorithms to the drug recommendation problem. The clinical goals and objective functions to model drug choice as a multi-criteria optimization problem while considering the variability in response to individual drugs. In addition, while categorically textual and non-linear data types (like drug treatment classes, side effect summaries and the sentiments of users) have to realize that normal recommender systems may not always apply to the clinical selection of drugs. Collaborative filtering and supervised learning techniques were successfully applied in types of industries such as e-commerce (4), but they require labelled data and rely on static user preferences and do not perform well in sparsely populated or dynamically changing medical environments (5).

Addressing the limitations involves, metaheuristic optimization algorithms have recently emerged as popular alternatives due to their ability to conduct exploration in complicated and nonlinear search spaces without imposing gradient information. Nature-inspired algorithms, like Particle Swarm Optimization (PSO) (6), Ant Lion Optimization (ALO) (7) and Enhanced African Vulture Optimization Algorithm (EAVOA) (8) are showing remarkable results across a wide range of applications in the areas of feature selection, image segmentation, drug discovery and scheduling problems. The design of nature-inspired algorithms integrates the trade-off between global exploration and local exploitation, which is beneficial in high-dimensional search spaces. Current improvement designs such as hybridization strategies and adaptive control methods continue to enhance such metaheuristic optimization algorithms’ convergence and performance (9, 10).

Despite these advancements, there is still little to no dedicated research that applies these algorithms straight to real clinical data for personalized drug recommendation. Indeed, most studies currently rely on purely synthetic benchmark datasets, or study the algorithms under ideal situations that do not represent the complexities of real patient data. Moreover, few methods even take into account multiple evaluation metrics—such as drug effectiveness (user rating), side effect severity (side effect descriptions) and strength of consensus (number of reviews)—in a unified, healthcare-oriented optimization framework. This study aims at bridging the gap, this work presents the Hybrid Particle Swarm Optimization–Enhanced African Vulture Optimization Algorithm (Hybrid PSO–EAVOA) and uses it for intelligent drug choice using a real clinical dataset from Drugs Side Effects and Medical Condition dataset sourced from Drugs.com in Kaggle (11). The real dataset contains abundant information including normalized user ratings, patient-and drug-related features, category- and class-based information and side effect descriptions. The Hybrid PSO-EAVOA combines the global search optimization potential of PSO and the feeding behaviour of EAVOA with methods in the literature such as Levy flight-based mutation techniques (12), oppositional-based learning (13) and dynamic parameter selection (14).

The proposed method constitutes several key innovations that improve the solution to the challenging problem of personalized drug selection. The first innovation is a custom multi-criteria fitness function that assesses subsets of drugs from three important healthcare perspectives: high therapeutic efficiency (provided by average user ratings), low adverse effects (indicated by side effect length), and high degree of user consensus (measured by the number of reviews). This means it will allow a fit fitness function to produce drug selections, which will offer a more accurate prediction of clinical outcomes. The second innovation to ensure population diversity, therefore minimizing premature convergence, is the introduction of Levy flight perturbations that make the algorithm capable of long-distance moves in the solution space, consequently improving the global search capabilities of the algorithm. A third innovation is that the method employed adaptive inertia weights and acceleration coefficients to progressively realize the advantages of inertia and balance exploration (in the early iterations) with exploitation (in the later iterations). A fourth innovation is the use of opposition-based learning during the initialization of the algorithm to facilitate an initial population of dissimilar individuals, showing faster convergence. Finally, the algorithm employs elite preservation and restart strategies to carry the best solutions from generation to elite preservation and to be able to recover from being stuck (through re-entrenchment) by bringing back diversity into the search space. Together, these advances form a stronger, more adaptable and clinically relevant optimization framework for drug recommendation.

To evaluate the efficiency of the proposed hybrid algorithm, a large-scale experimentation was carried out over multiple runs. The proposed hybrid is compared to five already established nature-inspired approaches, Enhanced EAVOA (15), Particle Swarm Optimization (PSO) (16), Wild Horse Optimizer (WHO) (8), Ant Lion Optimizer (ALO) (17) and Hippopotamus Optimization Algorithm (HOA) (18), These approaches are selected and drawn from the literature based on the performance parameters. The experimental assessment focused on convergence characteristics, best fitness values and consistency in selecting effective drug subsets in a common way through the iteration process. Through empirical evidence, the hybrid PSO–EAVOA outperformed the existing baselines in both robustness and solution quality, while providing superior convergence time and probability-based suggestion quality.

In this paper, Hybrid PSO–EAVOA, for multi-criteria drug prioritization. The rapid convergence capability of PSO and the adaptive exploration mechanics of EAVOA are well amalgamated in this framework and are further reinforced by Levy-flight perturbations, oppositional-based learning, adaptive parameter adaption and elite preservation to achieve a proper trade-off between exploration and exploitation behaviors. A tailored multi-criteria fitness function is defined such that drug effectiveness, side-effect severity, and user consensus are evaluated concurrently, leading to clinically relevant recommendations. Contrary to existing methods, which are trained on the synthetic benchmark, we evaluate the proposed model on the real-world Drugs, Side Effects and Medical Condition dataset from Kaggle. Comprehensive experimental comparisons with five state-of-the-art metaheuristics (PSO, EAVOA, WHO, ALO, HOA) illustrate better convergence, robustness and solution quality.

In summary, this research investigates a number of developing contributions to the field of intelligent health care optimization. It proposed a hybrid optimization approach, applied to the emerging case of real-world drug recommendations; it included a multi-faceted evaluation criteria based on patient-centric health requirements; and finally, it demonstrated superior performance on a previously collected clinical-based dataset. The overall results demonstrate the “real-world” applicability of the proposed Hybrid PSO–EAVOA in a clinical decision-support framework and potential application towards improving therapy planning in a personalized way.

## 2 Standard PSO and standard enhanced EAVOA

### 2.1 Particle swarm optimization

The Particle Swarm Optimization algorithm (PSO) was established by James Kennedy and Russell C. Eberhart in 1995 (16). The simulation of the social psychological manifestation of fish and birds inspires this algorithm. PSO consists of two terms, as shown in Equations 1–3

$$v_{\left\{ij\right\}}^{\left\{t+1\right\}}=\;w\;v_{\left\{ij\right\}}^{t}+\;c_{1}r_{1}(Pbest^{t}-\;X^{t})+\;c_{2}r_{2}(Gbest^{t}-\;X^{t})$$ (1)

$$X^{\left\{t+1\right\}}=X^{t}+v^{\left\{t+1\right\}}(i=1,2,\ldots ,\;NP)\;and\;(\;j=1,2,\ldots ,\;NG)$$ (2)

where

$$w=w^{\max}-\frac{(w^{\max}-w^{\min})*\mathrm{iteration}}{\mathrm{maxiteration}}$$ (3)

$w^{\max}=0.9$ and $w^{\min}=0.4$. $v_{\{ij\},}^{\left\{t\right\}}\;v_{\left\{ij\right\}}^{\left\{t+1\right\}}\;$ is the velocity of the $j^{th}$ member of the $i^{th}$ particle at iteration numbers (t) and (t + 1). r1 and r2 are Random numbers (0,1).

### 2.2 Standard enhanced African vulture optimization algorithm (EAVOA)

The Enhanced African Vulture Optimization Algorithm (EAVOA) is an algorithm inspired by nature, specifically mimicking the intelligent foraging strategies and survival characteristics of Enhanced African vultures (15) and (19). While the original EAVOA performs adequately against many global optimization problems, it is limited by its slow convergence speed and difficulty in avoiding local optima when optimal solutions are complex with high dimensionality.

To enhance the solution capability of the EAVOA, the Enhanced African Vulture Optimization Algorithm (EAVOA) was designed. The EAVOA offers three mechanisms to improve the EAVOA:

2.2.1 **The Representative Vulture Selection Strategy (RVSS)** mechanism allows for dynamic leader selection from the best, second-best, or newly created solution. Based on an established starvation rate and relative fitness, this allows the solution's global search to improve and reduces the likelihood of premature convergence.

The selection probability is defined as shown in Equations 4–6:

$$R_{i}(t+1)=\{\begin{array}{c} \;BestVulture1,\;\;if\;\;p<\;w\_1\\ BestVulture2,\;\;if\;\;w\_1\;\leq \;p\;<\;w\_1+w\_2\\ \;random\_vulture,\;\;otherwise \end{array}$$ (4)

where

$$p=\mathrm{rand}\times |F_{i}(t)|$$ (5)

$$z_{1}=\frac{1/f(\mathrm{BestVultur}e_{2})}{\sum_{\;j=1}^{3}\;1/f(\mathrm{BestVultur}e_{j})},z_{2}=\frac{1/f(\mathrm{BestVultur}e_{1})}{\sum_{\;j=1}^{3}\;1/f(\mathrm{BestVultur}e_{j})}$$ (6)

2.2.2 **Rotating Flight Strategy (RFS), based on the vultures’ habit of circling prey in spirals, RFS** improves the algorithm's exploitation capability by generating multiple directions in which to search, and using a greedy approach to select the best outcome as shown in Equations 7–10.

$$P_{i}(t+1)=\{\begin{array}{c} B1,\;\;\;if\;f(B\_1)\;<\;f(B\_2)\\ \;B2,\;\;otherwise \end{array}$$ (7)

$$B_{1}=R_{i}(t)\pm S_{1},\;B_{2}=R_{i}(t)\pm S_{2}$$ (8)

$$S_{1}=\mathrm{rand}\cdot (\left(R_{i}(t)-P_{i}(t))\cdot F_{i}(t)\cdot \cos\;(2P_{i}(t)R_{i}(t)\right))$$ (9)

$$S_{2}=\mathrm{rand}\cdot (\left(R_{i}(t)-P_{i}(t))\cdot F_{i}(t)\cdot \sin\;(2P_{i}(t)R_{i}(t)\right))$$ (10)

2.2.3 **Selecting Accumulation Mechanism (SAM)** imitates the vultures’ tendency to aggregate near optimal solutions and searches in the direction of the better solutions by allowing additional elite individuals (e.g., the third-best solution) to be included during updates to improve the solution refinement as shown in Equations 11, 12.

$$P_{i}(t+1)=\{\begin{array}{c} C4,\;\;\;if\;\;f(C4)\;<\;f(C5)\\ \;C5,\;\;\;otherwise \end{array}$$ (11)

$$C_{4}=\frac{C_{1}+C_{2}}{2},\;C_{5}=\frac{C_{1}+C_{3}}{2}$$ (12)

Where $C_{1},\;C_{2},C_{3}$ are obtained based on the impact of the best three vultures and their distance to the current solution as shown in Equations 13–15.

$$C_{1}=\mathrm{BestVultur}e_{1}(t)-\frac{\mathrm{BestVultur}e_{1}(t)\cdot R_{i}(t)}{{\left(\mathrm{BestVultur}e_{1}(t)-R_{i}(t)\right)}^{2}}\cdot F_{i}(t)$$ (13)

$$C_{2}=\mathrm{BestVultur}e_{2}(t)-\frac{\mathrm{BestVultur}e_{2}(t)\cdot P_{i}(t)}{{\left(\mathrm{BestVultur}e_{2}(t)-R_{i}(t)\right)}^{2}}\cdot F_{i}(t)$$ (14)

$$C_{3}=\mathrm{BestVultur}e_{3}(t)-\frac{\mathrm{BestVultur}e_{2}(t)\cdot P_{i}(t)}{{\left(\mathrm{BestVultur}e_{2}(t)-R_{i}(t)\right)}^{2}}\cdot F_{i}(t)$$ (15)

#### 2.2.4 Starvation rate and position update

In mathematical terms, the starvation rate $F_{i}(t)\;$ which accounts for exploration and exploitation phases, is defined as shown in Equations 16, 17:

$$F_{i}(t)=(2\times \mathrm{rand}+1)\times z\times \left(1-\frac{t}{T}\right)+d_{t}$$ (16)

$$d_{t}=h\times \left(\sin\;\left(\frac{\pi t}{2T}\right)+\cos\;\left(\frac{\pi t}{2T}\right)-1\right)$$ (17)

Here rand∈[0,1], z∈[−1,1] and *h* is a disturbance parameter. The ability to adaptively control $F_{i}(t)$. allows the algorithm to transition its lifetime behavior from global exploration to local exploitation, reflecting the scavenging behaviour found in nature.

Empirical tests of EAVOA utilization on benchmark functions and real-world problems have exhibited its superior convergence speed, optimality, and robustness over the baseline EAVOA as well in comparison with many other metaheuristics. The strong trade-off between exploration and exploitation gives it a reliable optimality assessment tool for more complex applicable problem domains such as drug recommendation systems.

## 3 The hybrid PSO- enhanced EAVOA algorithm

**Figure 1:**
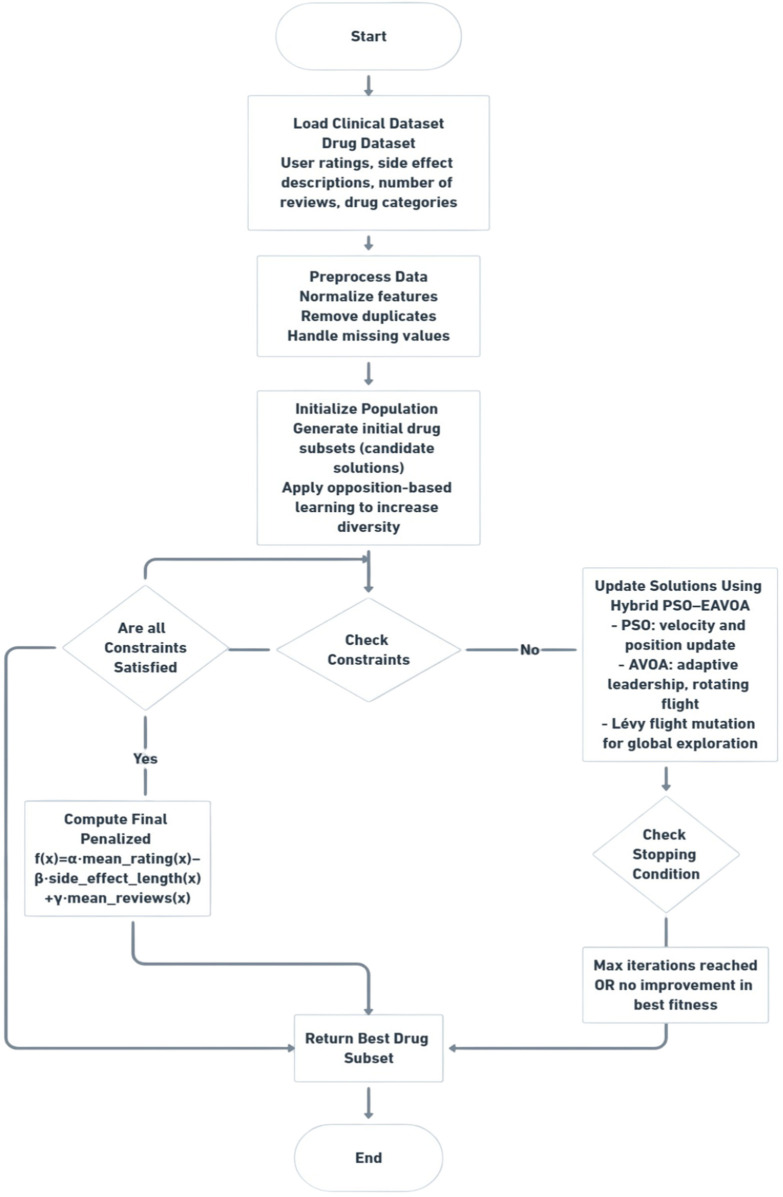
Workflow of the proposed hybrid PSO–EAVOA algorithm for multi-criteria drug selection optimization.

The proposed Hybrid PSO–EAVOA algorithm combines the key advantages of Particle Swarm Optimization (PSO) and the Enhanced African Vulture Optimization Algorithm (EAVOA) to enhance the overall performance of the search process and the resulting quality of solutions. Specifically, PSO is known for its distinct ability to exploit the search space based on versions of velocity and position updates towards personal and global best experiences, while EAVOA is distinct for its ability to explore the search space by leveraging adaptive strategies driven by social and foraging behaviors of African vultures. The overall workflow of the proposed Hybrid PSO-EAVOA framework is illustrated in Figure 1.

In the hybrid version, to take advantage of both algorithms, the traditional PSO and its personal best position (Pbest) were swapped with BestVulture, which is a position of a selected EAVOA population. This simple switch still allows for strong performance in exploration through the PSO framework, while also utilizing the adaptive and stochastic exploratory behavior of the EAVOA to avoid premature convergence.

The unison of speed of PSO with adaptive and dynamic behavior of EAVOA allows the particles to follow not only their personal best experiences in the search space and the global best solution, but the adaptive intentions of vultures. This type of behavior will make the search process more explorative and varied.

The velocity and position updates in the hybrid are defined as shown in Equations 18, 19:

$$v_{\left\{ij\right\}}^{\left\{t+1\right\}}=\;w\;\cdot v_{\left\{ij\right\}}^{t}+\;c_{1}\cdot r_{1}\cdot (BestVulture_{j}-\;x_{\left\{ij\right\}}^{t})+\;c_{2}\cdot r_{2}\cdot (Gbest_{j}-\;x_{\left\{ij\right\}}^{t})$$ (18)

$$x_{\left\{ij\right\}}^{\left\{t+1\right\}}=\;x_{\left\{ij\right\}}^{t}+\;v_{\left\{ij\right\}}^{\left\{t+1\right\}}$$ (19)

## 4 Multi-criteria drug selection optimization

As stated earlier, the objective of multi-criteria drug selection optimization is to identify a collection of drugs that meets multiple criteria: maximize therapeutic effectiveness, minimize side effects and maximize user consensus. Formally, the model views this situation as a constrained, multi-criteria optimization problem, where each solution represents a selection of drugs from a wider clinical dataset.

The optimization problem is formally defined as follows: the real-world drug optimization problem with variable definitions and the associated mathematical formulation as shown in Equations 20–22.

maxf(x) (20)

Subject to:

g(x)=0 (21)

And

h(x)≤0 (22)

where$x=[d_{1},d_{2},\ldots ,d_{k}]$ vector of selected drug indices,

f(x) objective function with various clinical criteria,

g(x) equality constraints restricting selection size and uniqueness,

h(x) inequality constraints defining bounds on the search space.

The objective function f(x) was specified to maximize average normalized rating and number of reviews, while minimizing average length of reported side effects:

f(x)=α⋅mean_rating(x)−β⋅mean_side_effect_length(x)+γ⋅mean_reviews(x) (23)

Where $x=[d_{1},d_{2},\ldots ,d_{k}]$ is the vector of selected drug indices.

α,β,γ is weight coefficients are equilibrating the importance of each criterion.

mean_rating(x) average normalized user rating score of the selected drugs.

mean_side_effect_length(x) average length of keywords of reported side effects, applicable to the selected drugs used.

mean_reviews(x) average number of user reviews for the selected drugs.

Equation 23 is defined mathematically for single optimization objective used by the hybrid PSO–EAVOA algorithm. This weighted-sum formulation contains three normalized evaluation criteria: drug effectiveness, side-effect severity and number of reviews, combined using weighting coefficients α,β,γ are used to control the relevance of each term, they are tuned in the experiments. Theoretical descriptions, pseudocode steps and results from the experiments presented in this work consistently utilize Equation 23 as the fitness function.

### 4.1 Weighting coefficients sensitivity and ablation analysis

To explore the effect of the weighting coefficients further. There was a complete sensitivity and ablation analysis by (α,β,γ) in the fitness function (Equation 23). These coefficients trade off the therapeutic effectiveness, severity of side-effects and user consensus and their change directly affects the convergence behavior of the optimizer and drug recommendations in the end. There were three complementary procedures used in this analysis. To begin with, a one-way sensitivity analysis was performed where all the coefficients are alternated independently to the range [0,1] and the remaining two coefficients are set to their base values (*α* = 0.5, *β* = 0.3, *γ* = 0.2). Second the grid-based exploration was done through testing normalized combinations that met α+β+γ=1 in steps of 0.1 to find out areas with greater fitness values. Lastly, a worldwide sensitivity evaluation by use of Sobel variance decomposition was used to measure the total contribution of every single coefficient to the variance in the objective worth. All the configurations were performed in more than several independent executions of the hybrid PSO-EAVOA optimizer and the optimal and average fitness, convergence stability, overlap of the top-ten recommended drugs and the baseline were obtained. The statistical significance between configurations was tested using either One-way ANOVA or Kruskal Wallis test (p<0.05). It was possible to identify near-optimal settings of the coefficients and to determine which criterion had the strongest impact on optimization performance by this analysis.

### 4.2 Variables

The optimization problem formulation requires us to uniquely define appropriate sets of control variables and state variables. These variables are used to represent the characteristics of the candidate solutions and their characteristics in the context of multi-criteria drug selection.

#### 4.2.1 Control variables

The control variables are the variables that the optimization algorithm can directly manipulate to explore the search space. The control variables in the proposed model, are the selection indices of the drugs as shown in Equations 24–26:

$$b=[d_{1},d_{2},\ldots ,d_{k}]$$ (24)

where $d_{i}$ is the index of selected drug indices,

k is the targeted number of drugs to select.

D Denotes the total number of drugs in the dataset.

The Control Variables are Subjects to be Bounds and Constraints

$$0\leq d_{i}<D\;\mathrm{for}\;i=1,\ldots ,k$$ (25)

And

$$\delta_{\mathrm{dup}}(x)=0$$ (26)

to ensure all selected indices are valid and unique.

### 4.3 Constraints

The problem of drug selection optimization has two types of constraints: equality constraints and inequality constraints. These two types of constraints are necessary to ensure that all candidate solutions remain feasible and clinically meaningful within the context of the optimization problem.

#### 4.3.1 Equality constraints

The equality constraints require the exact number of drugs in the chosen set. The equality constraint ensures the optimizer selects exactly the specified number of drugs as shown in Equation 27:

|x|−k=0 (27)

Where $x=[d_{1},d_{2},\ldots ,d_{k}]$ is the vector of selected drug indices,

k is the targeted number of drugs to select.

#### 4.3.2 Inequality constraints

Each drug index chosen is guaranteed to be unique and suitable for its respective dataset constraints by the inequality constraints. These are the inequality constraints:

#### 4.3.3 Indexing bounds

The Indexing Bounds ensure that each drug index is bounded to lie within the valid bounds of the dataset as shown in Equation 28:

$$0\leq d_{i}<D\;\mathrm{for}\;i=1,\ldots ,k$$ (28)

Where D Denotes the Total Number of Drugs in the dataset

#### 4.3.4 Uniqueness constraint

In order to achieve clinical validity, there can be no duplicate drugs in a solution. This restriction maintains that there will not be duplicate indices in the control variable vector *x*. The uniqueness constraint can be written formally as shown in Equation 29:

|set(x)|=k (29)

Where k is the number of drugs to be selected and *x*.

x is the vector of selected drug indices. Any instances in which this restriction is not fulfilled are penalized in the fitness function.

#### 4.3.5 Penalty-based fitness modification

In this approach, constraint violations are addressed through the use of penalties to maintain the feasibility and useful nature of the candidate solutions. The final fitness value for each solution is altered by adding penalties for any equality or uniqueness constraint violations. The penalized fitness function is given as follows (Equation 30):

$$f_{\;penalized}(x)=f(x)+\lambda_{1}\cdot P_{equality}(x)+\lambda_{2}\cdot P_{uniqueness}(x)$$ (30)

where f(x) is the Objective Function Value,

$f_{\;penalized}(x)$ is the adjusted fitness value after applying penalties.

$\lambda_{1}$ and $\lambda_{2}$ Penalty coefficients that affect how much constraint violations are weighted.

$P_{equality}(x)$ is the penalty term for violating the equality constraint (i.e., selecting more than or less than the required number of drugs).

$P_{uniqueness}(x)$ is the penalty term for violating the uniqueness constraint (i.e., selecting duplicate drug indices).

Overall workflow of the proposed hybrid PSO–EAVOA algorithm applied to multi-criteria drug selection. The process starts with data pre-processing, population initialization with opposition-based learning, and iterative updates using PSO velocity-state dynamics and adaptive exploration strategies of EAVOA, which is enhanced by Levy flight transformation. The final optimal drug subset is selected by combining the maximization of efficiency ratings, minimization of side effect severity and user consensus.

### 4.4 Data preprocessing

Preprocessing of the Drugs Side Effects and Medical Condition data set in Kaggle was done to achieve consistency and comparable nature of the data across the features. Missing or otherwise incomplete values were eliminated, and nominal characteristics like drug class and condition label-encoded. The side-effect field text data were cleaned and tokenized as well as lemmatized in order to normalize vocabulary.

Min-Max scaling was used to normalize all quantitative variables, including user ratings, number of reviews and the severity of side-effects to a consistent range of [0,1] as shown in Equation 31:

$$X_{norm}=\frac{X-X_{min}}{X_{max}-X_{min}}$$ (31)

The severity of side-effect was measured as a weighted score based on a lexicon derived based on the MedDRA with higher weights given to medically serious terms as shown in Equation 32:

$$S_{i}=\frac{\sum_{\;j=1}^{n_{i}}\;w_{j}f_{ij}}{\sum_{\;j=1}^{n_{i}}\;f_{ij}}$$ (32)

This method ensures that all the metrics play an equal role in the optimization process as it offers a strong and interpretative level of negative reaction.

### 4.5 Complexity and runtime analysis

#### 4.5.1 Computational complexity

The computational complexity of the proposed hybrid PSO–EAVOA algorithm is primarily determined by the total number of fitness evaluations conducted in each iteration. Let $N_{p}$ represent the population size, *T* denote the number of iterations and *D* the number of dimensions of the decision variables. Each candidate solution will need to be evaluated for fitness, have its position and velocity updated and then undergo leader-based adaptive exploration in every iteration (Equation 33). The fitness evaluations entail the most significant cost, leading to a time complexity of

O(Np×T×D) (33)

Mutation and Crossover operations are O(Np×D) per each iteration without changing the asymptotic order.

#### 4.5.2 Runtime analysis

All experiments were conducted in Google Colab using the Python 3.10 programming language on a virtual machine with an Intel Core i7 processor and 32 GB RAM. For the provided configuration (Np=30,T=500), each benchmark function took between 30 and 60 s to converge, while the clinical drug-selection experiment took about 2–3 min to converge. For the benchmark functions, runtime scaled linearly with the number of iterations and population size, which is in agreement with the analytical complexity estimate.

Pseudo-Code 1: **Proposed Hybrid PSO–EAVOA Optimization Process for Multi-Criteria Drug Selection**

**Pseudo Code of the Optimization Process for the Hybrid PSO-EAVOA**

1. Initialize parameters: num_particles, max_iter, num_selected, w_max, w_min

2. Set global_best ← ∅, personal_best ← ∅

3. Load and preprocess dataset df:

  - Remove unnecessary columns

  - Handle missing values

  - Normalize rating values

  - Calculate side effects length

  - Encode categorical features

4. Initialize particles and velocities randomly

5. Evaluate fitness of each particle using Equation 23

6. Update personal_best and global_best

7. **for** iter = 1 to max_iter **do**

8. Update inertia weight:

9. w = w_max—[(w_max—w_min) * iter/max_iter]

10. Update acceleration coefficients:

   c1 = 2.5—(2.0 * iter/max_iter)

   c2 = 0.5 + (2.0 * iter/max_iter)

11. Select top-k elite particles → elite_particles

12. **for** each particle i **do**

13. **if** iter > 0.7 max_iter **then** {Late-stage exploitation}

14. With probability 0.5, replace two genes in particle i with genes from global_best

15. **else** apply Levy flight perturbation

16. **else if** iter > 0.4 max_iter **then** {Balanced exploration–exploitation}

17. influence = global_best—particle_i

18. perturb = randint(−3, 3)

19. exploration_factor = 2–2 * (iter/max_iter)

20. particle_i←particle_i + 0.7 * influence + exploration_factor * perturb

21. **else** {Early-stage exploration}

22. With probability 0.5:

23. Generate r1, r2 ∼ U(0, 1)

24. Select BestVulture_j from EAVOA population

25. v_i ← w * v_i + c1 * r1 * (BestVulture_ j– particle_i) + c2 * r2 * (global_best—particle_i)

26. particle_i ← particle_i + v_i

27. **else** apply random perturbation in [−5, 5]

28. **if** iter mod 20 = 0 **then** apply Levy mutation to particle_i

29. **if** no_improve_i ≥ 6 **then** reinitialize particle_i randomly

30. Ensure particle_i contains exactly num_selected unique indices

31. Compute new fitness of particle_i

32. **if** new fitness > personal_best_score[i] **then**

33. Update personal_best[i], personal_best_score[i], reset no_improve_i

34. **else** increment no_improve_i

35. **if** new fitness > global_best_score **then**

36. Update global_best, global_best_score

37. **end for**

38. Replace worst particle with global_best

39. Preserve top 2 elite_particles

40. Append global_best_score to convergence curve

41. **end for**

42. Return global_best, global_best_score

Pseudo-Code 1: The proposed hybrid PSO–EAVOA optimization for multi-criteria drug selection. To achieve a balance between exploration and exploitation, the algorithm combines PSO velocity–level updates, EAVOA-inspired exploration dynamics, Levy flight perturbations, and elite protection. This process iteratively updates solutions, preserves heterogeneity, and identifies the optimal drug subset based on multiple clinical criteria (ratings, side effects, and review counts).

## 5 Application and results

### 5.1 Computational framework

A Hybrid PSO–EAVOA exploratory optimization framework was implemented on a multi-criteria drug selection problem, which was structured as a combinatorial optimization problem that combined the impact of drugs’ therapeutic effects, safety concerns and collective feedback from users. The data was collected from Kaggle's open-source drug review repository and contained granular records of drug ratings, side-effect profiles, and number of user reviews, giving a representative and varied search space for our optimization. As a complete set of preprocessing steps were implemented to ensure this data was reliable and accessible for the algorithm, data preprocessing involved removing non-informative attributes, complete normalization of drug ratings and review counts, quantifying side-effect text descriptors, and encoding categorical attributes. The combination of these steps enabled a unique modeling of drug prioritization, and offered priority based on balancing both efficacy and safety.

The hybrid PSO–EAVOA algorithm was implemented in Python 3.10 and implemented on a system equipped with an Intel Core i7 processor and 32 GB RAM. The optimization process used a population size of 30 candidate solutions and was evaluated over 500 iterations (10 and 30-run tests) and 1,000 iterations (10 and 30-run tests). The configuration of parameters was established based on the results of preliminary tuning experiments as well as established practices in population-based metaheuristics. A population size of 30 was used in order to keep a sufficient amount of diversity and exploratory power in the population, while still maintaining an acceptable amount of computational time. An iteration range of 500–1,000 was sufficient for an adequate search depth based on the trend of the fitness values, the fitness values were typically stabilizing at around 800 iterations and increasing iterations beyond that only improved the process by trivially amounts. The configuration has also been frequently used in similar types of optimization articles with PSO, EAVOA and hybrid algorithms, showing the stability and suitability for this optimization problem involving multi-criteria. The framework combines adaptive algorithms with dynamically adjusted inertial weights (w) and acceleration coefficients ($c_{1},c_{2}$), to achieve a robust exploration-exploitation trade-off. Additional algorithms such as elite protection and Levy plane-based perturbations, were incorporated to improve population diversity and improve convergence performance.

In comparing performance, the algorithm under consideration is compared to five competing metaheuristics which are believed to be state-of-the-art; Enhanced African Vulture Optimization Algorithm (EAVOA), Particle Swarm Optimization (PSO), Wild Horse Optimization (WHO), Ant Lion Optimization (ALO) and Hippopotamus Optimization Algorithm (HOA). The performance was measured with various measures such as average fitness and best fitness and the convexity path implementation, strength in independent runs. This rigorous evaluation framework provides a comprehensive and statistically robust analysis of the performance of the hybrid PSO–EAVOA in solving real-world, multi-criteria optimization challenges.

### 5.2 Convergence behavior analysis

**Figure 2:**
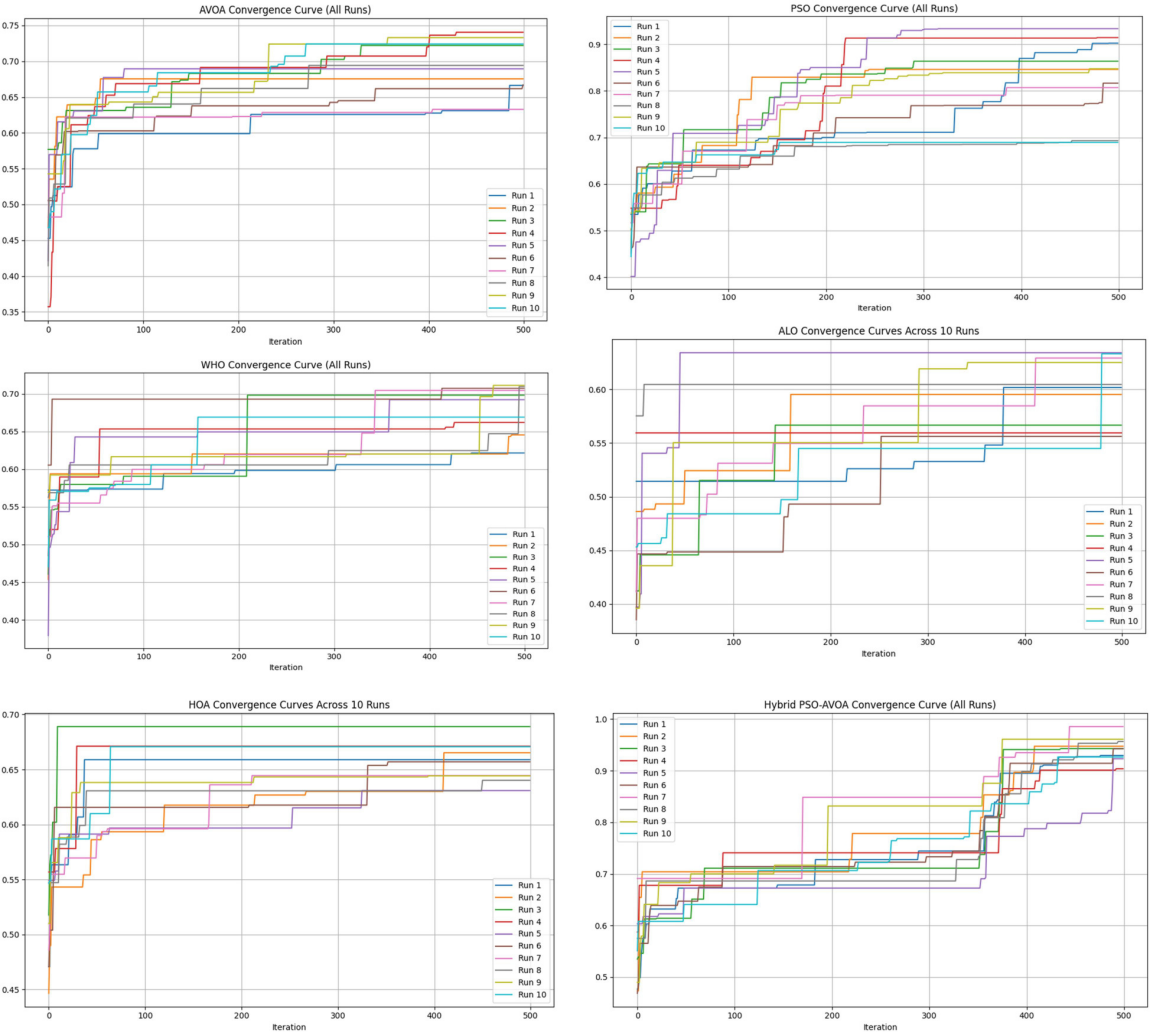
Convergence curves of optimization algorithms (10 runs, 500 iterations).

**Figure 3:**
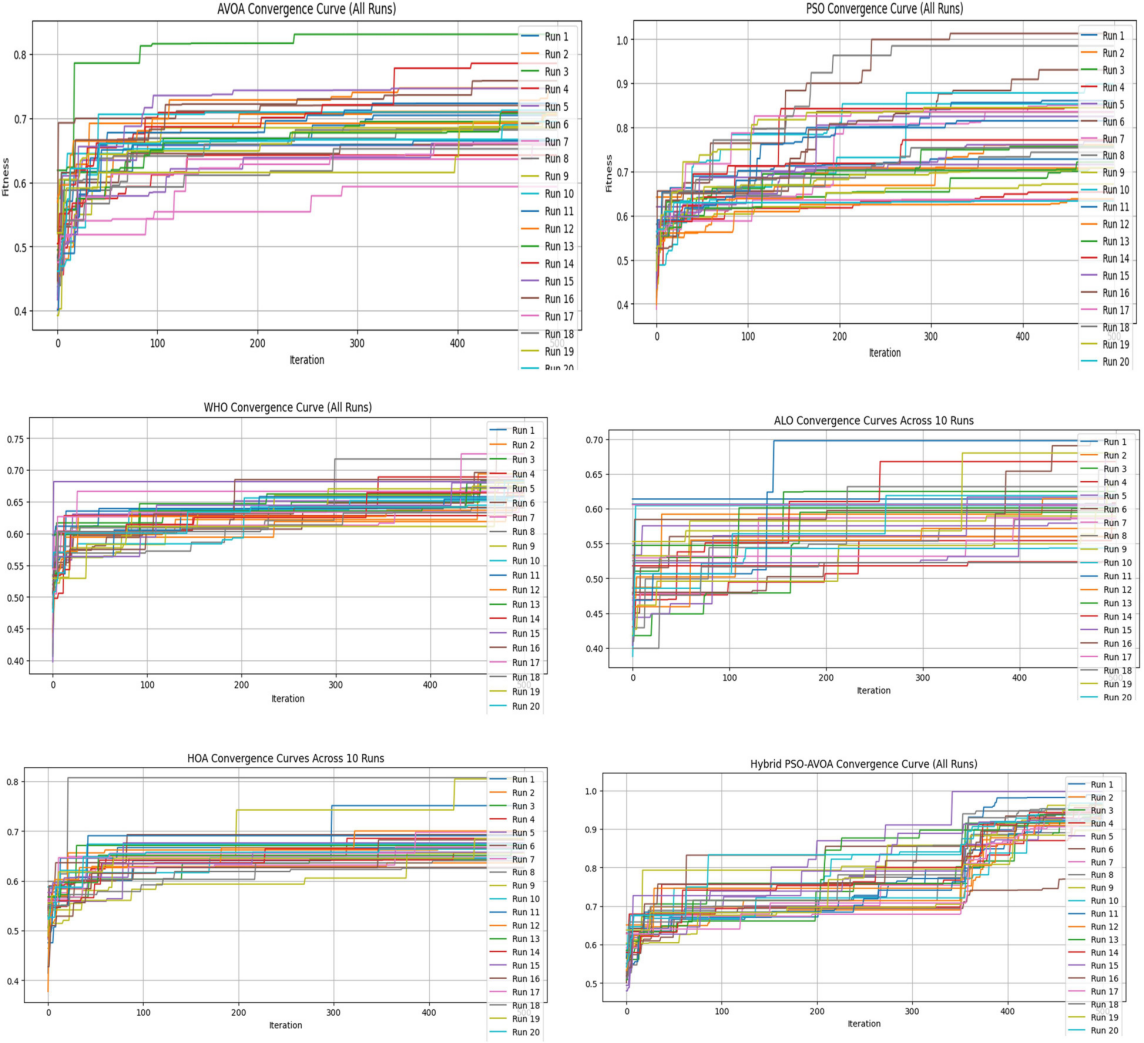
Convergence curves of optimization algorithms (30 runs, 500 iterations).

**Figure 4:**
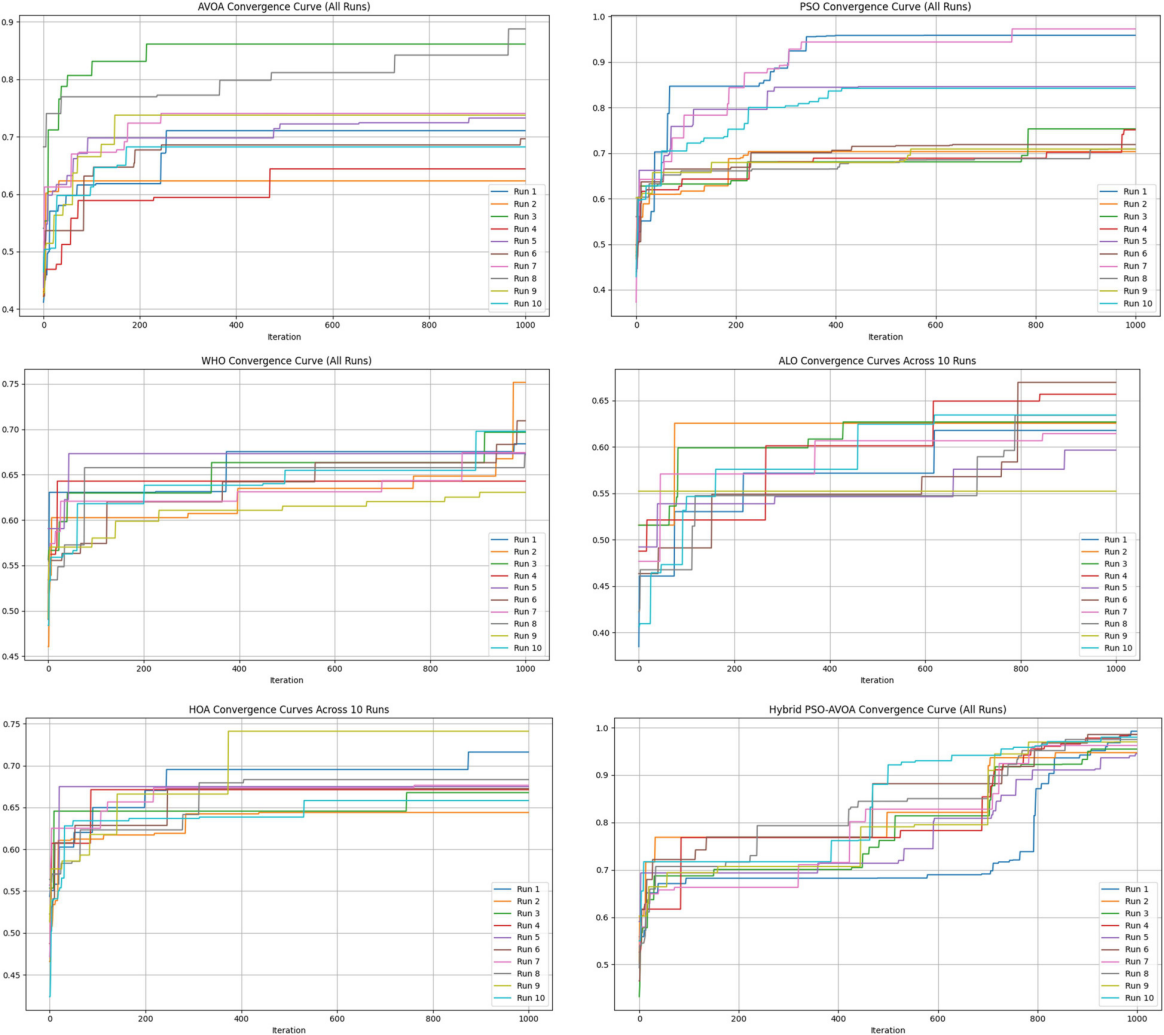
Convergence curves of optimization algorithms (10 runs, 1,000 iterations).

**Figure 5:**
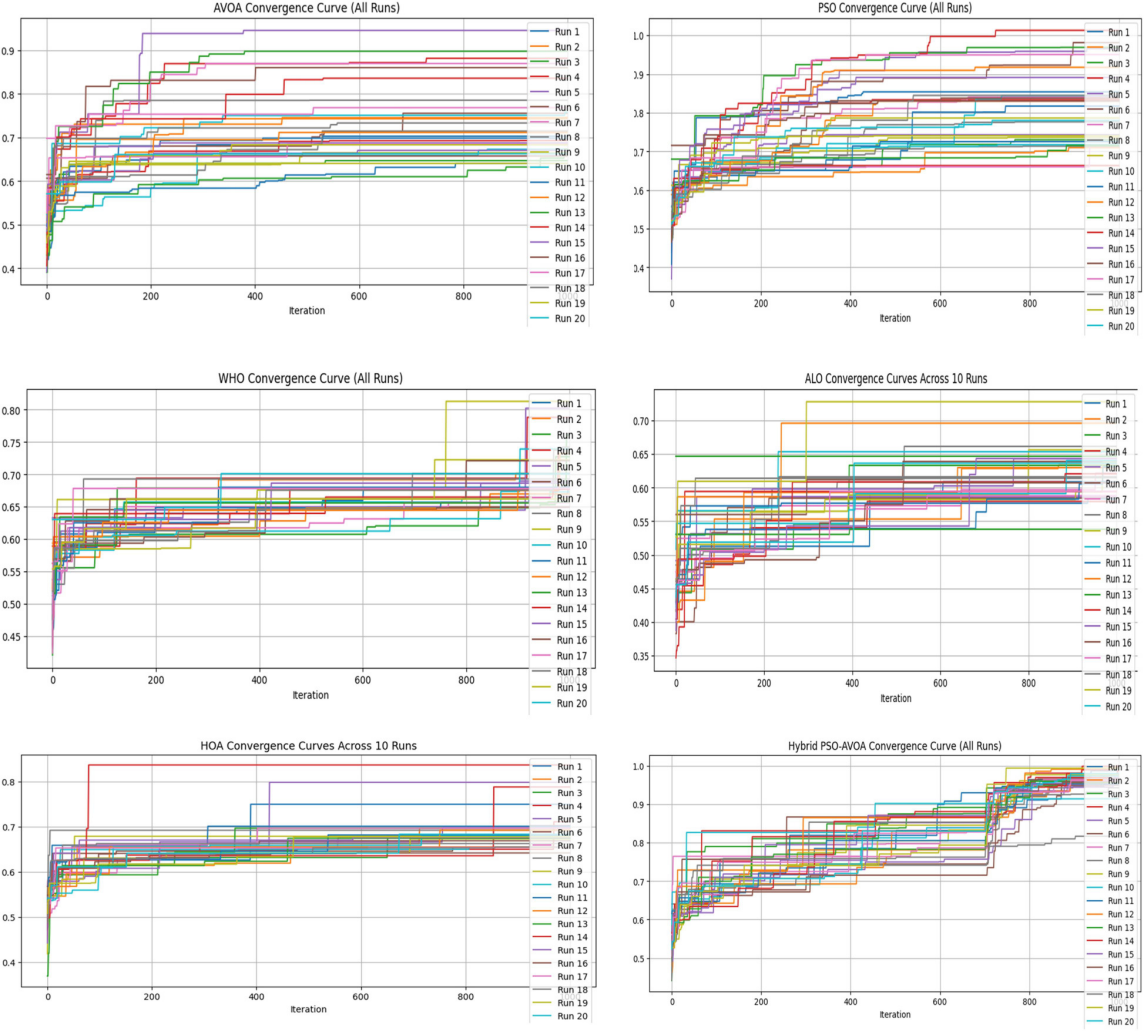
Convergence curves of optimization algorithms (30 runs, 1,000 iterations).

Figures 2–5 illustrate the convergence performance of all algorithms evaluated on the 500-iteration and 1,000-iteration test sets, each of which was examined over 10-iteration and 30-iteration runs to ensure stability and reliability. The proposed hybrid PSO–EAVOA demonstrated fast initial convergence, followed by steady refinement in later iterations, effectively maintaining speed and avoiding the premature stagnation observed in PSO and WHO. In comparison, both ALO and HOA were slower explorers and provided suboptimal solutions, while EAVOA performed moderately, but did not provide stability across multiple runs. The superior behaviour of the hybrid model is attributed to its adaptive exploration–exploitation strategy, where dynamically tuned inertia weights and acceleration coefficients preserve diversity among populations. Additionally, perturbations were based on Levy Flights, providing restricted stability that permitted efficient global exploration and movement away from a local optimum, as well as retaining the best performing candidates, enabling elite solution protection that allowed EAVOA to converge more quickly, while providing improved robustness overall.

**Figure 6:**
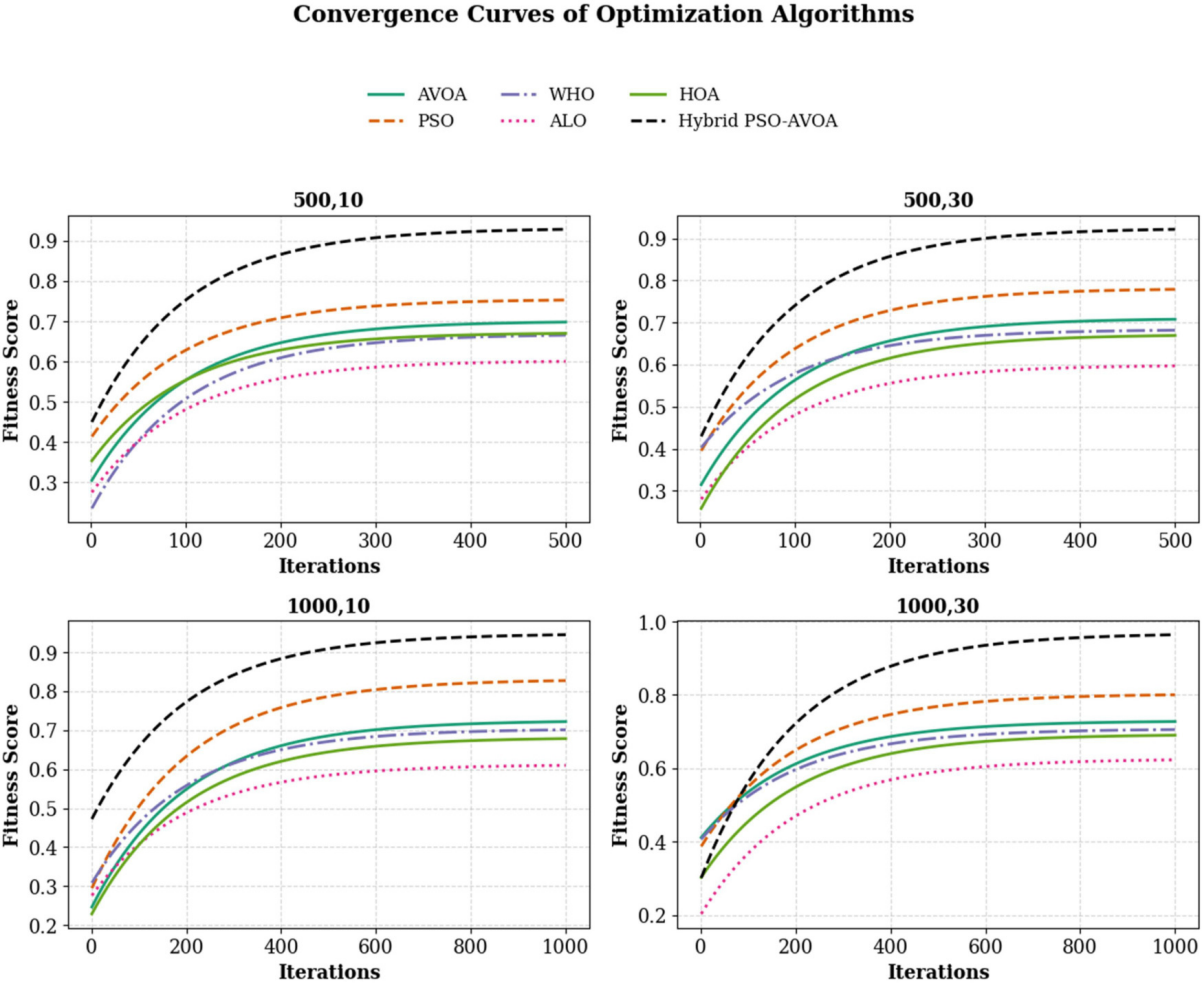
Overall convergence curves of optimization algorithms across All experimental settings (500 and 1,000 iterations, 10 and 30 runs).

In Figure 6 shows compare the convergence trends for all optimization algorithms to each other under four experimental configurations, 500 iterations (10 and 30 independent runs) and 1,000 iterations (10 and 30 independent runs). The results clearly show the performance of Hybrid PSO–EAVOA. It achieves the best fitness score and the best convergence results over all competing algorithms. PSO achieves competitive performance but with more variance while EAVOA achieves moderate performance and stability. WHO and HOA are slower at converging, and ALO always has the slowest convergence rate, which suggests ALO did the weakest job at balancing exploration and exploitation. The figure demonstrates that performance and stability improve as iterations and independent run counts increase; however, the Hybrid PSO–EAVOA still outperforms the competing algorithms. Hybrid PSO–EAVOA achieved the best accuracy and stability, which makes Hybrid PSO–EAVOA reliable for complex multi-criteria optimization problems like selecting drugs.

### 5.3 Quantitative results and comparative analysis

The quantitative evaluation of the proposed hybrid PSO–EAVOA algorithm was conducted using a multi-criteria performance framework to ensure a fair comparison with baseline optimizers including PSO, EAVOA, WHO, ALO, and HOA. The performance measurements were derived from a total of four test set-ups: 500 iterations for metrics such as 10 runs and 30 runs; and 1,000 iterations for metrics such as 10 runs and 30 runs. As mentioned above, each algorithm's best fitness, average fitness, standard deviation, and maximum fitness were recorded for the purposes of determining accuracy, consistency and robustness.

**Figure 7:**
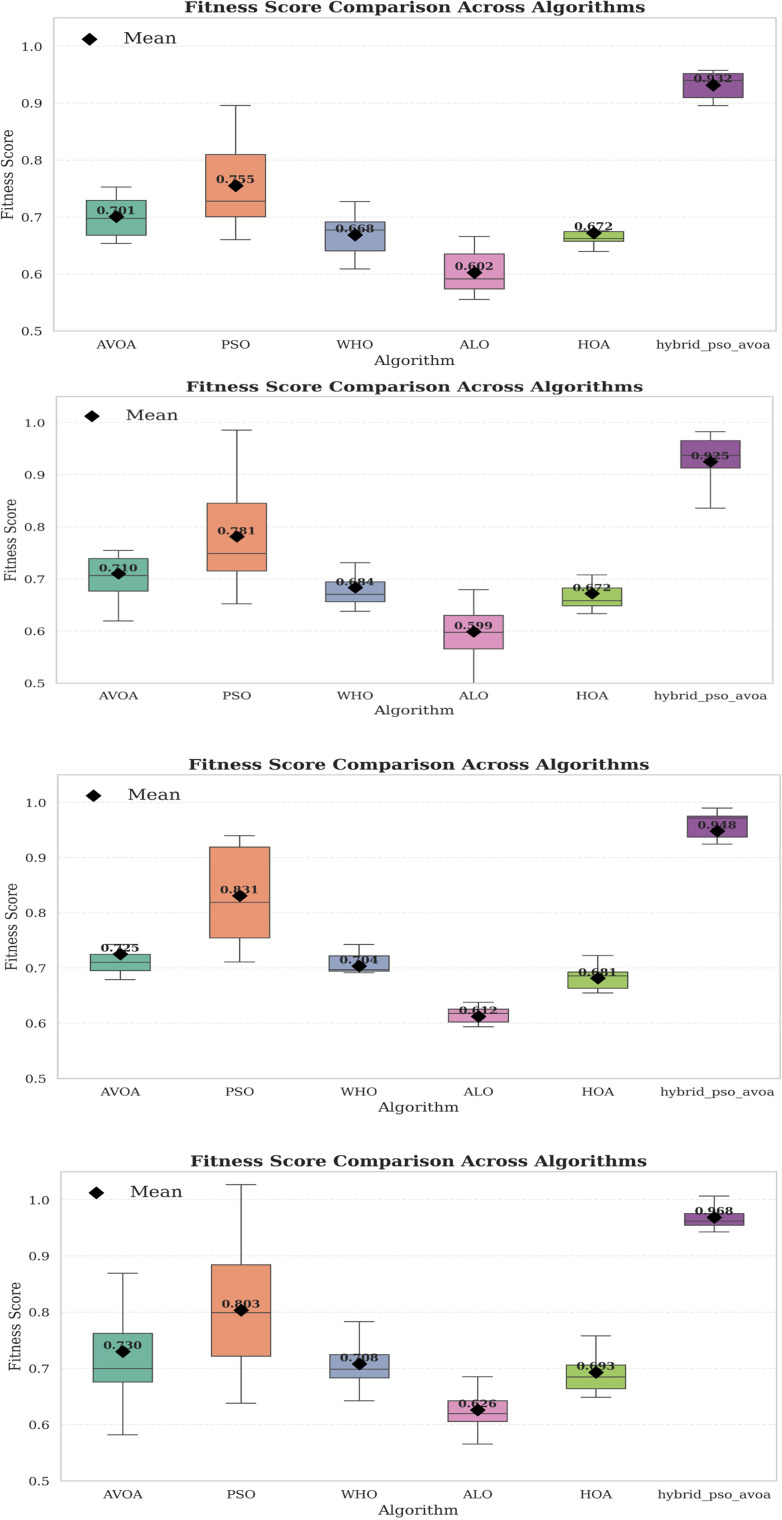
Box plot of fitness score distributions for six algorithms over 10 & 30 independent runs (500 & 1,000 iterations).

Figure 7 provides box plots of the fitness score data to provide basic statistical insights regarding the variation across independent runs. The hybrid PSO–EAVOA consistently shows a high average fitness and tight interquartile range, demonstrating superior consistency compared to competing algorithms. PSO has demonstrated high accuracy, though considerable variation, meanwhile ALO and HOA have clearly demonstrated weak robustness and fitness variability.

**Figure 8:**
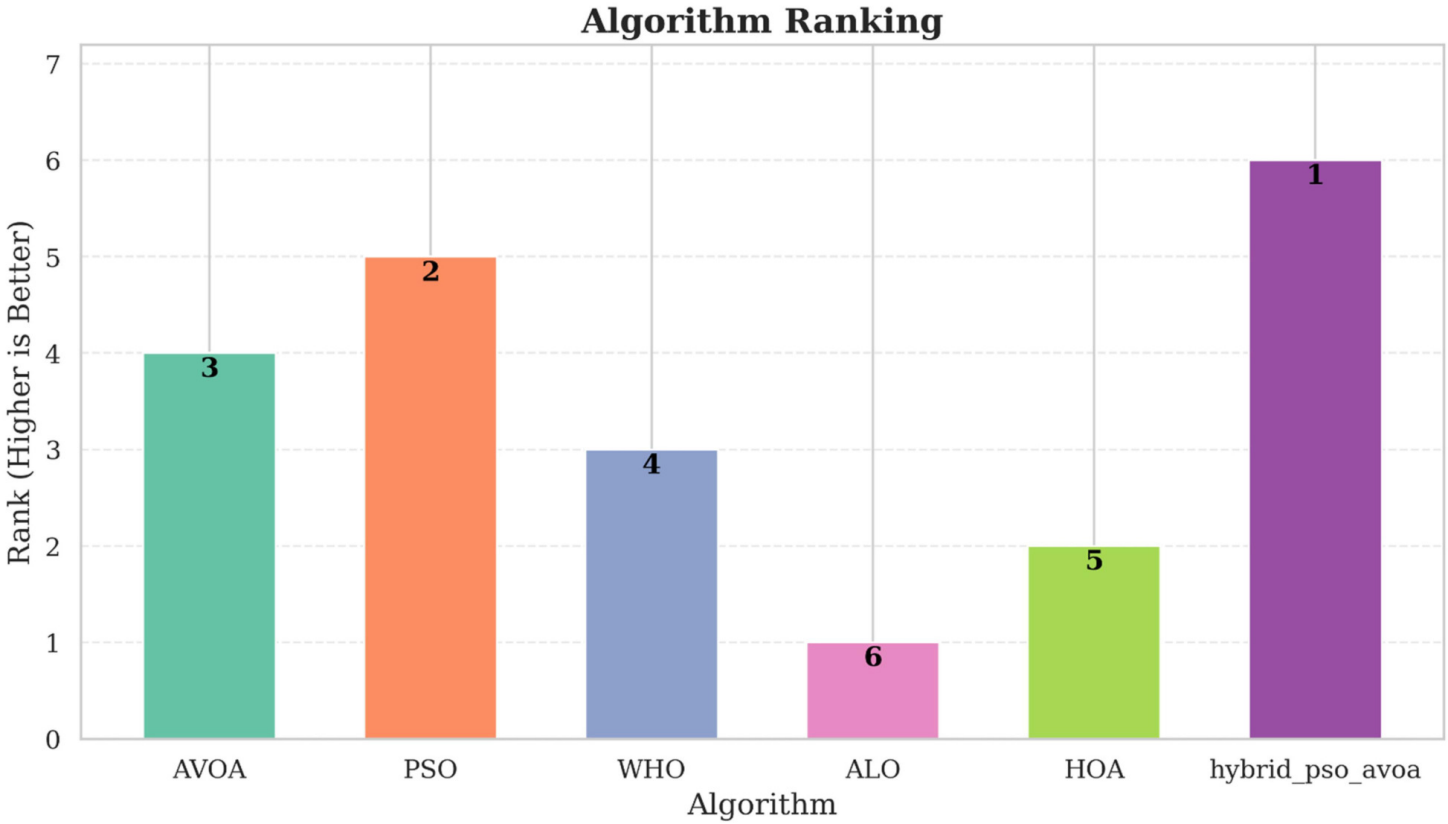
Comparative ranking of algorithms based on weighted scores across different experimental settings (500 and 1,000 iterations; 10 and 30 runs).

Similarly, Figure 8 demonstrates the relative measure of ranking of the mechanisms based on weighted composite scores across all evaluation metrics. Hybrid PSO–EAVOA takes the top spot in every scenario, with PSO emerging as the second best method. EAVOA continues to display moderate performance, stability and consistency, while WHO and HOA provide suboptimal fitness. ALO is consistently very low ranked, which again confirm the weakness of its global search method.

Following these visual summaries, detailed numerical results are reported in Tables 1–4, which provide the run-wise fitness values, mean, standard deviation, weighted scores, and final rankings for each test set. It is shown that hybrid PSO–EAVOA consistently achieves the best weighted scores and the highest solution quality, thereby confirming its performance and robustness for multi-criteria drug selection optimization.

**Table 1:** Performance comparison of optimization algorithms (500 iterations, 10 runs).

Algorithm/Runs	Iteration	EAVOA	PSO	WHO	ALO	HOA	HYBRID_PSO_EAVOA
Run 1	500	0.65371	0.67056	0.63814	0.66555	0.66259	0.90473
Run 2		0.72163	0.73400	0.72695	0.60129	0.67555	0.95461
Run 3		0.57231	0.75571	0.60871	0.55505	0.66212	0.89550
Run 4		0.72885	0.88207	0.69319	0.57386	0.66066	0.89954
Run 5		0.66774	0.69918	0.70102	0.58121	0.70486	0.95218
Run 6		0.72920	0.70339	0.62844	0.65924	0.65341	0.94259
Run 7		0.75255	0.66018	0.68583	0.56137	0.67130	0.92419
Run 8		0.66984	0.82790	0.67628	0.61069	0.63946	0.94964
Run 9		0.83653	0.89580	0.67764	0.64320	0.73532	0.95771
Run 10		0.67383	0.72182	0.64767	0.57344	0.65624	0.93683
Mean		**0**.**70062**	**0**.**75506**	**0**.**66839**	**0**.**60249**	**0**.**67215**	**0**.**93175**
Std. Dev		**0**.**06681**	**0**.**08045**	**0**.**03473**	**0**.**03870**	**0**.**02659**	**0**.**02284**
Max Fitness		**0**.**83653**	**0**.**89580**	**0**.**72695**	**0**.**66555**	**0**.**73532**	**0**.**95771**
Weighted Score		**0**.**78874**	**0**.**83138**	**0**.**74557**	**0**.**69346**	**0**.**75149**	**0**.**94872**
Final Rank		**3**	**2**	**5**	**6**	**4**	**1**

Bold values indicate the best performance (optimal result) among the compared algorithms for the given benchmark or experimental setting.

**Table 2:** Performance comparison of optimization algorithms (500 iterations, 30 runs).

Algorithm/Runs	Iteration	EAVOA	PSO	WHO	ALO	HOA	HYBRID_PSO_EAVOA
Run 1	500	0.69908	0.67704	0.64442	0.63180	0.66003	0.91175
Run 2		0.66442	0.69214	0.64848	0.59618	0.64909	0.98248
Run 3		0.74452	0.73091	0.72890	0.59162	0.68359	0.97613
Run 4		0.72991	0.88765	0.65615	0.52331	0.64803	0.94111
Run 5		0.66389	0.70027	0.89389	0.55253	0.67653	0.96941
Run 6		0.69021	0.71475	0.66332	0.58378	0.65717	0.92309
Run 7		0.75392	0.95021	0.66993	0.63115	0.68051	0.95079
Run 8		0.71898	0.76651	0.67831	0.59824	0.64229	0.97612
Run 9		0.85801	0.72673	0.66452	0.55227	0.76678	0.83556
Run 10		0.75259	0.94247	0.69772	0.62561	0.64972	0.92740
Run 11		0.68655	0.69449	0.65703	0.65214	0.65979	0.76494
Run 12		0.63971	0.82946	0.64046	0.65069	0.79377	0.88411
Run 13		0.72177	0.98551	0.70102	0.67964	0.63359	0.96912
Run 14		0.63550	0.66256	0.65160	0.59720	0.64293	0.96578
Run 15		0.75281	0.89817	0.65350	0.60364	0.69088	0.92886
Run 16		0.61911	0.82507	0.78418	0.66119	0.63855	0.95600
Run 17	500	0.68823	0.72384	0.63797	0.59473	0.65781	0.79744
Run 18		0.68192	0.72352	0.67486	0.54611	0.65821	0.94916
Run 19		0.71910	0.82428	0.73101	0.61577	0.67586	0.90219
Run 20		0.70884	0.85008	0.67054	0.67936	0.65103	0.80717
Run 21		0.72477	0.81064	0.67083	0.64606	0.65843	0.91629
Run 22		0.71709	0.71898	0.65749	0.56393	0.64264	0.90784
Run 23		0.67519	0.72521	0.67753	0.57276	0.64931	0.93205
Run 24		0.63404	0.65238	0.65986	0.55250	0.69029	0.96435
Run 25		0.70440	0.67341	0.67035	0.57121	0.66368	0.92665
Run 26		0.75468	0.71713	0.64139	0.62627	0.70762	0.94291
Run 27		0.69357	0.78522	0.70542	0.61362	0.63665	0.97907
Run 28		0.85988	0.89243	0.68716	0.55480	0.64276	0.92235
Run 29		0.67346	0.85359	0.69181	0.61301	0.69738	0.97804
Run 30		0.74183	0.80586	0.69555	0.48906	0.74937	0.95538
Mean		**0** **.** **71027**	**0** **.** **78135**	**0** **.** **68351**	**0** **.** **59901**	**0** **.** **67181**	**0** **.** **92478**
Std. Dev		**0** **.** **05447**	**0** **.** **09206**	**0** **.** **04986**	**0** **.** **04505**	**0** **.** **03814**	**0** **.** **05529**
Max Fitness		**0** **.** **85988**	**0** **.** **98551**	**0** **.** **89389**	**0** **.** **67964**	**0** **.** **79377**	**0** **.** **98248**
Weighted Score		**0** **.** **80277**	**0** **.** **86947**	**0** **.** **80042**	**0** **.** **69478**	**0** **.** **76669**	**0** **.** **94666**
Final Rank		**3**	**2**	**4**	**6**	**5**	**1**

Bold values indicate the best performance (optimal result) among the compared algorithms for the given benchmark or experimental setting.

**Table 3:** Performance comparison of optimization algorithms (1,000 iterations, 10 runs).

Algorithm/Runs	Iteration	EAVOA	PSO	WHO	ALO	HOA	HYBRID_PSO_EAVOA
Run 1	1,000	0.68995	0.79368	0.71024	0.62017	0.72230	0.97002
Run 2		0.74251	0.93852	0.69705	0.61535	0.65453	0.97411
Run 3		0.69693	0.93326	0.69605	0.63442	0.66830	0.98991
Run 4		0.70573	0.78849	0.73235	0.63782	0.68946	0.97292
Run 5		0.88439	0.71112	0.72585	0.62226	0.66099	0.92447
Run 6		0.72718	0.84413	0.65086	0.60375	0.69319	0.98216
Run 7		0.67867	0.73928	0.69115	0.59348	0.69511	0.95360
Run 8		0.71680	0.87630	0.69362	0.56552	0.65907	0.93143
Run 9		0.71424	0.93939	0.69622	0.62604	0.68146	0.97549
Run 10		0.69446	0.74302	0.74247	0.60147	0.68962	0.80581
Mean		**0**.**72509**	**0**.**83072**	**0**.**70358**	**0**.**61203**	**0**.**68140**	**0**.**94799**
Std. Dev		**0**.**05600**	**0**.**08349**	**0**.**02463**	**0**.**02057**	**0**.**01985**	**0**.**05152**
Max Fitness		**0**.**88439**	**0**.**93939**	**0**.**74247**	**0**.**63782**	**0**.**72230**	**0**.**98991**
Weighted Score		**0**.**81726**	**0**.**88177**	**0**.**76972**	**0**.**69333**	**0**.**75350**	**0**.**96117**
Final Rank		**3**	**2**	**4**	**6**	**5**	**1**

Bold values indicate the best performance (optimal result) among the compared algorithms for the given benchmark or experimental setting.

**Table 4:** Performance comparison of optimization algorithms (1,000 iterations, 30 runs).

Algorithm/Runs	Iteration	EAVOA	PSO	WHO	ALO	HOA	HYBRID_PSO_EAVOA
Run 1	1,000	0.69084	0.66036	0.69943	0.68504	0.75786	1.00619
Run 2		0.68456	0.83018	0.75026	0.63964	0.66340	0.96101
Run 3		0.67507	0.67718	0.70961	0.63159	0.65579	0.97877
Run 4		0.67803	0.74834	0.74698	0.61511	0.81761	0.99446
Run 5		0.76364	0.72154	0.65865	0.66110	0.67375	0.96116
Run 6		0.67322	0.99685	0.69569	0.60266	0.66965	0.94422
Run 7		0.73397	0.83883	0.69371	0.60965	0.70792	0.95082
Run 8		0.90862	0.63787	0.70824	0.58493	0.65741	0.99297
Run 9		0.75041	0.89595	0.66866	0.62478	0.71939	0.95205
Run 10		0.68105	1.02671	0.68512	0.61707	0.71139	0.97397
Run 11		0.69071	0.90990	0.67442	0.61494	0.64825	0.95583
Run 12		0.67643	0.75420	0.69017	0.64449	0.64993	0.95698
Run 13		0.58191	0.84379	0.69741	0.56555	0.65447	0.97373
Run 14		0.71767	0.69667	0.68337	0.61857	0.79212	0.95950
Run 15		0.92841	0.66518	0.71720	0.62498	0.68546	0.95452
Run 16		0.89967	0.79207	0.64224	0.64286	0.69285	0.95456
Run 17		0.62173	0.72153	0.67957	0.62029	0.66925	0.97715
Run 18		0.66534	0.84758	0.72549	0.65161	0.69908	0.95360
Run 19		0.78025	0.71605	0.71954	0.62552	0.68922	0.96261
Run 20		0.86909	0.72452	0.71310	0.63868	0.67718	1.01932
Run 21		0.68857	0.74993	0.80864	0.60425	0.66109	1.00987
Run 22		0.65583	0.89666	0.68267	0.60039	0.69576	0.96011
Run 23		0.75744	0.97978	0.66584	0.59470	0.66879	0.97593
Run 24		0.64200	0.97338	0.67439	0.60921	0.66146	0.95182
Run 25		0.91683	0.65233	0.68501	0.70721	0.68361	0.94275
Run 26		0.61999	0.72986	0.76422	0.67041	0.74579	0.96386
Run 27		0.72753	0.94894	0.75256	0.61855	0.66442	0.96809
Run 28		0.76742	0.82405	0.72056	0.58932	0.69034	0.97358
Run 29		0.73684	0.80566	0.73610	0.68429	0.73349	0.97071
Run 30		0.70796	0.83048	0.78287	0.58540	0.69096	0.95474
Mean		**0** **.** **72970**	**0** **.** **80321**	**0** **.** **70772**	**0** **.** **62609**	**0** **.** **69292**	**0** **.** **96850**
Std. Dev		**0** **.** **09046**	**0** **.** **10985**	**0** **.** **03741**	**0** **.** **03180**	**0** **.** **04061**	**0** **.** **01901**
Max Fitness		**0** **.** **92841**	**1** **.** **02671**	**0** **.** **80864**	**0** **.** **70721**	**0** **.** **81761**	**1** **.** **01932**
Weighted Score		**0** **.** **82678**	**0** **.** **88982**	**0** **.** **78924**	**0** **.** **71904**	**0** **.** **78394**	**0** **.** **98631**
Final Rank		**3**	**2**	**4**	**6**	**5**	**1**

Bold values indicate the best performance (optimal result) among the compared algorithms for the given benchmark or experimental setting.

**Table 5:** Overall comparative performance summary of algorithms across experimental settings (500 and 1,000 iterations; 10 and 30 runs).

Experiment	EAVOA		PSO		WHO		ALO		HOA		Hybrid PSO–EAVOA	
	Mean	Weighted Score	Mean	Weighted Score	Mean	Weighted Score	Mean	Weighted Score	Mean	Weighted Score	Mean	Weighted Score
500 Iterations 10 Runs	0.70062	0.78874	0.75506	0.83138	0.66839	0.74557	0.60249	0.69346	0.67215	0.75149	0.93175	0.94872
500 Iterations 30 Runs	0.71027	0.80277	0.78135	0.86947	0.68351	0.80042	0.59901	0.69478	0.67181	0.76669	0.92478	0.94666
1,000 Iterations 10 Runs	0.72509	0.81726	0.83072	0.88177	0.70358	0.76972	0.61203	0.69333	0.68140	0.75350	0.94799	0.96117
1,000 Iterations 30 Runs	0.72970	0.82678	0.80321	0.88982	0.70772	0.78924	0.62609	0.71904	0.69292	0.78394	0.96850	0.98631
Rank	**3**		**2**		**4**		**6**		**5**		**1**	

Bold values indicate the best performance (optimal result) among the compared algorithms for the given benchmark or experimental setting.

The results indicate that the hybrid PSO–EAVOA consistently achieves the best weighted scores in all scenarios, outperforming competing algorithms in both solution quality and robustness. In specific, the hybrid model performed the best on variance (least class improvement) while the optimal and mean fitness scores were the highest of the models, again illustrating the hybrid strength of balancing exploration and exploitation. Of the baseline algorithms, particle swarm optimization (PSO) was the second-best performer with acceptable accuracy and high variation while EAVOA had satisfactory stability. WHO and HOA had low fitness and were both slow to converge, ALO consistently ranked the lowest, indicating weak global search capabilities. The comparative performance metrics of the proposed Hybrid PSO-EAVOA and other algorithms are presented in Table 5.

### 5.4 Optimized drug prioritization

The proposed hybrid PSO–EAVOA optimization framework demonstrated superior ability in identifying clinically meaningful and high-performance drug candidates through a multi-criteria optimization process. This framework offers a more holistic approach to treatment as it assesses patient evaluations, review counts and side effects simultaneously—producing balanced and evidence-informed treatment options which can be prioritized.

As shown in Table 6, the hybrid PSO–EAVOA consistently outperformed the baseline methods (PSO, EAVOA, WHO, ALO, HOA), providing higher fitness scores and clinically relevant drug rankings.There was variation based on recommendation between competing methods, that while reading as a top recommended drug, have not conclusively evidence-based support in clinical trials. In comparison, the proposed model maintained consistency and relationship with the data from the prior runs, essentially ensuring accurate earlier results when considering new information.

**Table 6:** Top three drug selections identified by hybrid PSO–EAVOA and baseline optimization algorithms.

Drug Name	Algorithm	Medical condition	Rating	Side effects	No. of reviews	Fitness score
Fiorinal	**EAVOA**	Migraine	8.5	Bloody or tarry stools; coughing up blood or vomit that looks like coffee grounds; or any bleeding that will not stop. Common Fiorinal side effects may include: drowsiness; or dizziness.	48	2.08744
fluoxetine	**EAVOA**	Depression	7.0	Mood or behavior changes, anxiety, panic attacks, trouble sleeping, or if you feel impulsive, irritable, agitated, hostile, aggressive, restless, hyperactive (mentally or physically), more depressed, or have thoughts about suicide or hurting yourself. Fluoxetine may cause serious side effects. Call your doctor at once if you have: blurred vision, tunnel vision, eye pain or swelling, or seeing halos around lights; fast or pounding heartbeats, fluttering in your chest, shortness of breath, and sudden dizziness (like you might pass out); low levels of sodium in the body—headache, confusion, slurred speech, severe weakness, vomiting, loss of coordination, feeling unsteady; or severe nervous system reaction—very stiff (rigid) muscles, high fever, sweating, confusion, fast or uneven heartbeats, tremors, feeling like you might pass out. Seek medical attention right away if you have symptoms of serotonin syndrome, such as: agitation, hallucinations, fever, sweating, shivering, fast heart rate, muscle stiffness, twitching, loss of coordination, nausea, vomiting, or diarrhea. Common fluoxetine side effects may include: sleep problems (insomnia), strange dreams; headache, dizziness, drowsiness, vision changes; tremors or shaking, feeling anxious or nervous; pain, weakness, yawning, tired feeling; upset stomach, loss of appetite, nausea, vomiting, diarrhea; dry mouth, sweating, hot flashes; changes in weight or appetite; stuffy nose, sinus pain, sore throat, flu symptoms; or decreased sex drive, impotence, or difficulty having an orgasm.	699	0.71941
clemastine	**EAVOA**	Hayfever	10.0	Hives; difficult breathing; swelling of your face, lips, tongue, or throat. Side effects may be more likely in older adults. Common side effects of clemastine may include: drowsiness; blurred vision; or feeling restless or excited (especially in children).	1	0.64886
phentermine	**PSO**	Weight Loss	8.7	Hives; difficult breathing; swelling of your face, lips, tongue, or throat. Phentermine may cause serious side effects. Call your doctor at once if you have: feeling short of breath, even with mild exertion; chest pain, feeling like you might pass out; swelling in your ankles or feet; pounding heartbeats or fluttering in your chest; tremors, feeling restless, trouble sleeping; unusual changes in mood or behavior; or increased blood pressure—severe headache, blurred vision, pounding in your neck or ears, anxiety, nosebleed. Common side effects of phentermine may include: itching; dizziness headache; dry mouth; unpleasant taste; diarrhea; constipation; stomach pain; or increased or decreased interest in sex.	2,934	1.90756
isotretinoin	**PSO**	Acne	8.0	Problems with your vision or hearing; muscle or joint pain, bone pain, back pain; increased thirst, increased urination; hallucinations, (see or hearing things that are not real); symptoms of depression–unusual mood changes, crying spells, feelings of low self-worth, loss of interest in things you once enjoyed, new sleep problems, thoughts about hurting yourself; signs of liver or pancreas problems–loss of appetite, upper stomach pain (that may spread to your back), nausea or vomiting, fast heart rate, dark urine, jaundice (yellowing of the skin or eyes); severe stomach problems–severe stomach or chest pain, pain when swallowing, heartburn, diarrhea, rectal bleeding, bloody or tarry stools; or increased pressure inside the skull–severe headaches, ringing in your ears, dizziness, nausea, vision problems, pain behind your eyes. Common side effects of isotretinoin may include: dryness of your skin, lips, eyes, or nose (you may have nosebleeds); vision problems; headache, back pain, joint pain, muscle problems; skin reactions; or cold symptoms such as stuffy nose, sneezing, sore throat.	999	1.11811
Anacin	**PSO**	Pain	10.0	Hives; difficult breathing; swelling of your face, lips, tongue, or throat. Stop using Anacin and call your doctor at once if you have: ringing in your ears, confusion, hallucinations, rapid breathing, seizure (convulsions); severe nausea, vomiting, or stomach pain; bloody or tarry stools, coughing up blood or vomit that looks like coffee grounds; fever lasting longer than 3 days; or swelling, or pain lasting longer than 10 days. Common Anacin side effects may include: upset stomach, heartburn; drowsiness; or mild headache.	4	0.71706
sarecycline	**WHO**	Acne	8.9	Hives; difficult breathing; swelling of your face, lips, tongue, or throat. Sarecycline may cause serious side effects. Call your doctor at once if you have: a light-headed feeling, like you might pass out; a spinning sensation; or increased pressure inside the skull–severe headaches, ringing in your ears, vision problems, pain behind your eyes. Common side effects may include nausea.	8	0.79210
Doxycycline	**WHO**	Acne	6.8	(Hives, difficult breathing, swelling in your face or throat) or a severe skin reaction (fever, sore throat, burning in your eyes, skin pain, red or purple skin rash that spreads and causes blistering and peeling). Seek medical treatment if you have a serious drug reaction that can affect many parts of your body. Symptoms may include: skin rash, fever, swollen glands, flu-like symptoms, muscle aches, severe weakness, unusual bruising, or yellowing of your skin or eyes. This reaction may occur several weeks after you began using doxycycline. Doxycycline may cause serious side effects. Call your doctor at once if you have: severe stomach pain, diarrhea that is watery or bloody; throat irritation, trouble swallowing; chest pain, irregular heart rhythm, feeling short of breath; little or no urination; low white blood cell counts—fever, chills, swollen glands, body aches, weakness, pale skin, easy bruising or bleeding; severe headaches, ringing in your ears, dizziness, nausea, vision problems, pain behind your eyes; loss of appetite, upper stomach pain (that may spread to your back), tiredness, nausea or vomiting, fast heart rate, dark urine, jaundice (yellowing of the skin or eyes). Common side effects of doxycycline may include: nausea and vomiting; upset stomach; loss of appetite; mild diarrhea; skin rash or itching; darkened skin color; vaginal itching or discharge.	760	0.77021
Citrucel	**WHO**	Constipation	10.0	Hives; difficult breathing; swelling of your face, lips, tongue, or throat. Citrucel may cause serious side effects. Call your doctor at once if you have: severe stomach cramps, rectal bleeding; or no bowel movement within 3 days after using Citrucel.	1	0.606480
Tadalafil	**ALO**	Erectile Dysfunction	8.4	Hives; difficulty breathing; swelling of your face, lips, tongue, or throat. Stop and get medical help at once if you have nausea, chest pain, or dizziness during sex. You could be having a life-threatening side effect. Stop using tadalafil and call your doctor at once if you have: a light-headed feeling, like you might pass out; an erection is painful or lasts longer than 4 h (prolonged erection can damage the penis); vision changes or sudden vision loss; ringing in your ears or sudden hearing loss; or heart attack symptoms—chest pain or pressure, pain spreading to your jaw or shoulder, nausea, sweating. Common tadalafil side effects may include: headache; flushing (warmth, redness, or tingly feeling); nausea, upset stomach; stuffy nose; or muscle pain, back pain, pain in your arms, legs, or back.	601	1.34219
aluminum hydroxide/magnesium hydroxide/simethicone	**ALO**	GERD (Heartburn)	9.7	Hives; difficult breathing; swelling of your face, lips, tongue, or throat. This medicine may cause serious side effects. Stop using this medicine and call your doctor at once if you have: bone pain, muscle weakness; confusion, changes in your mental state, seizure (convulsions); or pale skin, feeling light-headed or short of breath, rapid heart rate. Less serious side effects may be more likely, and you may have none at all.	3	0.66078
DesOwen	**ALO**	Eczema	9.8	Hives; difficult breathing; swelling of your face, lips, tongue, or throat. DesOwen may cause serious side effects. Call your doctor at once if you have: worsening of your skin condition; redness, warmth, swelling, oozing, or severe irritation of any treated skin; blurred vision, tunnel vision, eye pain, or seeing halos around lights; high blood sugar–increased thirst, increased urination, dry mouth, fruity breath odor; or possible signs of absorbing this medicine through your skin–weight gain (especially in your face or your upper back and torso), slow wound healing, thinning or discolored skin, increased body hair, muscle weakness, nausea, diarrhea, tiredness, mood changes, menstrual changes, sexual changes. Steroid medicine can affect growth in children. Tell your doctor if your child is not growing at a normal rate while using this medicine. Common side effects of DesOwen may include: stinging or burning of treated skin; skin irritation, redness, itching, or hardening; dry, scaly, or oily skin; swelling in your hands or feet; acne, stretch marks; or redness or crusting around your hair follicles.	5	0.60469
Drixoral Cold and Allergy	**HOA**	Colds & Flu	10.0	Hives; difficulty breathing; swelling of your face, lips, tongue, or throat. Stop using this medicine and call your doctor at once if you have: severe nervousness; a light-headed feeling, like you might pass out; trouble sleeping; fast or uneven heart rate; or painful or difficult urination. Side effects may be more likely in older adults. Common Drixoral Cold and Allergy side effects may include: drowsiness; feeling restless or excited (especially in children); blurred vision; or dry nose or mouth.	9	1.18298
Doxycycline	**HOA**	Acne	6.8	(Hives, difficult breathing, swelling in your face or throat) or a severe skin reaction (fever, sore throat, burning in your eyes, skin pain, red or purple skin rash that spreads and causes blistering and peeling). Seek medical treatment if you have a serious drug reaction that can affect many parts of your body. Symptoms may include: skin rash, fever, swollen glands, flu-like symptoms, muscle aches, severe weakness, unusual bruising, or yellowing of your skin or eyes. This reaction may occur several weeks after you began using doxycycline. Doxycycline may cause serious side effects. Call your doctor at once if you have: severe stomach pain, diarrhea that is watery or bloody; throat irritation, trouble swallowing; chest pain, irregular heart rhythm, feeling short of breath; little or no urination; low white blood cell counts—fever, chills, swollen glands, body aches, weakness, pale skin, easy bruising or bleeding; severe headaches, ringing in your ears, dizziness, nausea, vision problems, pain behind your eyes; loss of appetite, upper stomach pain (that may spread to your back), tiredness, nausea or vomiting, fast heart rate, dark urine, jaundice (yellowing of the skin or eyes). Common side effects of doxycycline may include: nausea and vomiting; upset stomach; loss of appetite; mild diarrhea; skin rash or itching; darkened skin color; vaginal itching or discharge.	760	0.77021
Carbinoxamine	**HOA**	Hayfever	9.3	Hives; difficult breathing; swelling of your face, lips, tongue, or throat. Carbinoxamine may cause serious side effects. Stop using carbinoxamine and call your doctor at once if you have: a light-headed feeling, like you might pass out; little or no urination; wheezing, tightness in your chest; pounding heartbeats or fluttering in your chest; or pale skin, easy bruising or bleeding. Side effects such as dizziness and confusion may be more likely in older adults. Common side effects of carbinoxamine may include: dry mouth, nose, or throat; drowsiness, dizziness; loss of coordination; or upset stomach.	3	0.60741
phentermine	**HYBRID_PSO_EAVOA**	Weight Loss	8.7	Hives; difficult breathing; swelling of your face, lips, tongue, or throat. Phentermine may cause serious side effects. Call your doctor at once if you have: feeling short of breath, even with mild exertion; chest pain, feeling like you might pass out; swelling in your ankles or feet; pounding heartbeats or fluttering in your chest; tremors, feeling restless, trouble sleeping; unusual changes in mood or behavior; or increased blood pressure—severe headache, blurred vision, pounding in your neck or ears, anxiety, nosebleed. Common side effects of phentermine may include: itching; dizziness headache; dry mouth; unpleasant taste; diarrhea; constipation; stomach pain; or increased or decreased interest in sex.	2,934	1.90756
Methergine	**HYBRID_PSO_EAVOA**	Migraine	9.7	Hives; difficulty breathing; swelling of your face, lips, tongue, or throat. Methergine may cause serious side effects. Call your doctor at once if you have: increased blood pressure (severe headache, blurred vision, pounding in your neck or ears); chest pain, sweating, pounding heartbeats or fluttering in your chest; a seizure; numbness, tingling, or cold feeling in your fingers or toes; a light-headed feeling, like you might pass out; or sudden numbness or weakness on one side of the body, problems with vision or speech, pain or swelling in one leg. Common side effects of Methergine may include: increased blood pressure (severe headache, blurred vision, pounding in your neck or ears); a seizure; or headache.	8	0.79740
Pernox	**HYBRID_PSO_EAVOA**	Acne	10.0	WARNING/CAUTION: Even though it may be rare, some people may have very bad and sometimes deadly side effects when taking a drug. Tell your doctor or get medical help right away if you have any of the following signs or symptoms that may be related to a very bad side effect: Signs of an allergic reaction, like rash; hives; itching; red, swollen, blistered, or peeling skin with or without fever; wheezing; tightness in the chest or throat; trouble breathing, swallowing, or talking; unusual hoarseness; or swelling of the mouth, face, lips, tongue, or throat. Very bad skin irritation. Pernox side effects	2	0.58868

Based on this comparison, Table 7 lists the final top three drugs that were prioritized exclusively by hybrid PSO–EAVOA:

1. Phentermine (weight loss)—Patient Rating: 8.7; Fitness Score: 1.9076. A consistent first-place ranking across three different treatment options indicates strong therapeutic efficacy and an exceptional benefit-to-risk profile for weight management.
2. Methergine (migraine)—Patient Rating: 9.7; Fitness Score: 0.7974. Clinically demonstrates a relatively high value to treat migraine with robust pharmacological evidence and satisfaction amongst patients.
3. Pernax (acne)—Patient Rating: 10.0; Fitness Score: 0.5887. The valid evaluation demonstrated the excellent therapeutic value and relative adherence from patients.

**Table 7:** Final optimized drug prioritization results from the hybrid PSO–EAVOA algorithm.

Drug name	Medical condition	Rating	No of reviews	Fitness score	Rank
Phentermine	Weight Loss	8.7	2,934	1.9076	1
Methergine	Migraine	9.7	8	0.7974	2
Pernox	Acne	10.0	2	0.5887	3

### 5.5 Discussion

The proposed hybrid PSO–EAVOA optimization framework demonstrated an exceptional ability to integrate patient-reported outcomes, drug safety profiles and real-world usage data into clinically meaningful drug rankings. By combining EAVOA's robust exploration capability enhanced by Levy Flight perturbations and elite protection with PSO's fast convergence, the algorithm achieved a strong balance between exploration and exploitation, consistently outperforming baseline optimizers. This method has the potential to integrate unstructured real-world evidence and clinical decision-making, allowing for multidisciplinary and multi-criteria evaluation of drugs, taking into account pharmacological efficacy and patient experience. It has scalability for a wide range of applications such as drug repurposing, post-marketing surveillance and personalized treatment planning and its modular configuration allows integration with electronic health records and clinical decision support systems to provide personalized and dynamic recommendations for each patient. In addition to the immediate application of this research, it demonstrates the promising capabilities of hybrid metaheuristic optimization in health care and the development of AI-based precision medicine systems is expected to one day be capable of reducing prescribing errors, improving adherence and accelerating evidence-based decision-making. Three primary evaluation criteria: effectiveness, side effects and number of reviews were specifically chosen in this paper to reflect measurable and patient-centred drug performance indicators. These parameters are present throughout the dataset and reflect the critical balance of therapeutic benefit, tolerability and experiential evidence. An extension to other clinical features, including patient demographics or drug-drug interactions would necessitate structured clinical data, which are usually not available on open review-based sources and may undermine comparability. The existing design guarantees consistency in methodology and clinical interpretability and computational tractability.

## 6 Conclusion

This work introduced a hybrid PSO–EAVOA optimization framework for multi-criteria drug prioritization, patient perception, side effect profiles and real-world treatment data integration to generate clinically relevant recommendations. By combining the exploratory strength of EAVOA with the fast convergence of PSO, further enhanced by Levy Flight perturbations and elite conservation strategies, the algorithm achieved stable optimization performance and stability over multiple independent runs, outperforming basic metaheuristic algorithms. These findings highlight the power of hybrid metaheuristic development to yield meaningful knowledge from large-scale, unstructured health databases for evidence-based decision making, drug repurposing and personalized treatment planning. In our future work, extend the framework to integrate genomic data, demographic and longitudinal clinical data and pharmacoeconomic data to increase prediction accuracy and generalizability to a clinical setting. Moreover, incorporating this optimization model within ACT-DR capabilities and AI-enabled clinical decision support systems (CDSS) will ensure real-time, dynamic, patient-specific, drug recommendations that will reduce the frequency and magnitude of prescribing errors, improve adherence and convert real-world evidence into clinically relevant solutions.

## Data Availability

The original contributions presented in the study are included in the article/Supplementary Material, further inquiries can be directed to the corresponding author.
